# Alopurinol Versus Trimetazidina para o Tratamento da Angina: Ensaio Clínico Randomizado

**DOI:** 10.36660/abc.20230659

**Published:** 2024-07-25

**Authors:** Tainá Viana, Rodrigo Morel Vieira de Melo, Diogo Freitas Cardoso Azevedo, Clara Salles Figueiredo, Gustavo Santana, Luanna Mota Damasceno, Luisa Latado, Ludmila Tambuque, Raissa Barreto, Luiz Carlos Santana Passos

**Affiliations:** 1 Universidade Federal da Bahia Salvador BA Brasil Universidade Federal da Bahia, Salvador, BA – Brasil; 2 Ana Nery Hospital Salvador BA Brasil Ana Nery Hospital, Salvador, BA – Brasil

**Keywords:** Alopurinol, Trimetazidina, Isquemia Miocárdica, Angina Pectoris

## Abstract

**Fundamento:**

Recentemente, foi demonstrado que o alopurinol, um inibidor da xantina oxidase, possui propriedades cardiovasculares e anti-isquêmicas e pode ser uma opção de agente antianginoso metabólico.

**Objetivo:**

O objetivo do presente estudo foi avaliar o efeito antianginoso do alopurinol como terceiro medicamento para pacientes com doença arterial coronariana (DAC) estável.

**Métodos:**

Trata-se de um ensaio clínico randomizado entre 2018 e 2020 incluindo pacientes com DAC que mantiveram angina apesar da otimização inicial com betabloqueadores e bloqueadores dos canais de cálcio. Os indivíduos foram randomizados 1:1 para 300 mg de alopurinol 2 vezes ao dia ou 35 mg de trimetazidina 2 vezes ao dia. O desfecho principal foi a diferença no domínio da frequência da angina do Questionário de Angina de Seattle (QAS-FA). Foram considerados estatisticamente significativos valores de probabilidade (p) < 0,05.

**Resultados:**

Foram incluídos 108 pacientes na fase de randomização, com 54 (50%) no grupo alopurinol e 54 (50%) no grupo trimetazidina. Seis (5,6%) indivíduos, 3 de cada grupo, foram perdidos no seguimento para o desfecho primário. Nos grupos de alopurinol e trimetazidina, as pontuações medianas do QAS-FA foram 50 (30,0 a 70,0) e 50 (21,3 a 78,3), respectivamente. Em ambos os grupos, a pontuação do QAS-FA melhorou, mas a mediana da diferença em relação à linha de base foi menor no grupo alopurinol (10 [0 a 30] versus 20 [10 a 40]; p < 0,001), assim como a média da diferença na pontuação total do QAS (12,8 ± 17,8 versus 21,2 ± 15,9; p = 0,014).

**Conclusão:**

Tanto o alopurinol quanto a trimetazidina melhoraram o controle dos sintomas de angina; no entanto, a trimetazidina apresentou um ganho maior em relação à linha de base. **Registro Brasileiro de Ensaios Clínicos – Número de Registro RBR-5kh98y**

**Figura Central:**
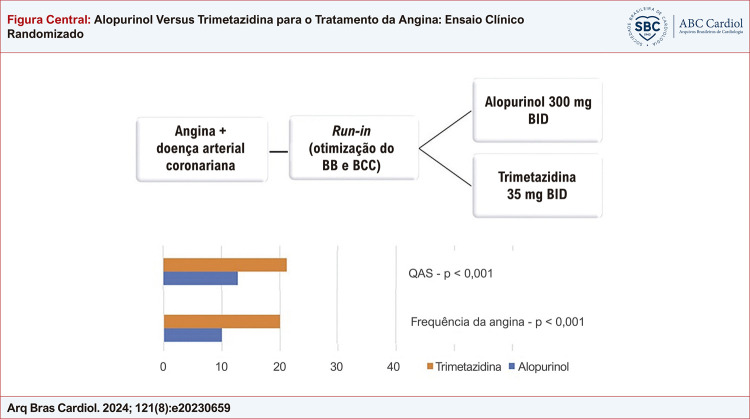
: Alopurinol Versus Trimetazidina para o Tratamento da Angina: Ensaio Clínico Randomizado

Alopurinol e trimetazidina melhoraram o controle dos sintomas de angina; no entanto, a trimetazidina levou a ganhos maiores em relação à linha de base. BB: betabloqueador; BCC: bloqueador dos canais de cálcio; BID: 2 vezes ao dia; QAS: Questionário de Angina de Seattle.

## Introdução

Com os avanços no tratamento de fatores de risco para a doença aterosclerótica, vários estudos têm demonstrado a eficácia dos tratamentos clínicos como a escolha inicial para doença arterial coronariana (DAC).^1 , 2^ As prioridades neste contexto são o controle dos fatores de risco e a melhora sintomática da angina.^3^

As diretrizes atuais recomendam o uso de betabloqueadores e bloqueadores dos canais de cálcio como medicamentos iniciais para o alívio dos sintomas de angina.^3^ Os agentes de segunda linha incluem trimetazidina, ivabradina e nitratos de ação prolongada. O alopurinol, um inibidor da xantina oxidase, demonstrou propriedades cardiovasculares e anti-isquêmicas.^4 - 7^ Em um estudo anterior, 300 mg de alopurinol 2 vezes ao dia aumentaram o tempo até a depressão do segmento ST e o tempo total no teste ergométrico.^8^

Entretanto, estudos que avaliaram o efeito do alopurinol na DAC utilizaram desfechos laboratoriais ou alterações em exames diagnósticos como desfecho primário.^4 - 8^ A prevalência de angina ou eventos cardiovasculares foi avaliada apenas como desfecho secundário; portanto, é necessário realizar estudos que avaliem, de maneira primária e sistemática, o efeito do medicamento na angina, que é o sintoma de maior impacto em pacientes com DAC estável.

O objetivo do estudo ATTRACT (Alopurinol versus Trimetazidina como Terceiro Medicamento para o Tratamento da Angina: Ensaio Clínico Randomizado) é comparar o alopurinol versus a trimetazidina como terceiro medicamento para controle da angina em pacientes com DAC e angina estável refratária às doses máximas toleradas de betabloqueadores e bloqueadores dos canais de cálcio

## Métodos

Trata-se de um ensaio clínico randomizado e unicêntrico que incluiu pacientes com sintomas de angina de um ambulatório especializado em DAC entre 2018 e 2020.

### Pacientes

Foram convidados a participar pacientes maiores de 18 anos com DAC estável diagnosticada por meio de cateterismo cardíaco que revelasse pelo menos 1 artéria coronária epicárdica com estenose superior a 70%, atendidos no ambulatório de um hospital de referência. A angiografia coronária foi realizada no contexto de síndrome coronariana aguda (SCA) prévia ou no contexto de doença coronária estável com elevada probabilidade de DAC ou sintomas persistentes. Todos os pacientes forneceram consentimento informado por escrito.

Os pacientes receberam tratamento clínico otimizado com um betabloqueador na dose máxima tolerada e um antagonista dos canais de cálcio diidropiridínico.

Os critérios de exclusão foram SCA nos últimos 3 meses, revascularização miocárdica cirúrgica ou percutânea programada, obstrução de tronco de coronária esquerda > 50%, angina assintomática após otimização clínica inicial, disfunção hepatocelular, doença renal crônica com depuração de creatinina menor que 30 ml/min/1,73 m^2^, artrite gotosa que justificasse o uso do alopurinol e recusa em participar do estudo e/ou assinar o termo de consentimento livre e esclarecido.

### Delineamento do estudo

Os pacientes foram submetidos a um período de 1 semana de *run-in* com o uso de um agente betabloqueador combinado com um antagonista dos canais de cálcio do tipo diidropiridínico em doses otimizadas. Os medicamentos foram acrescentados ou, quando o medicamento já estava em uso, as doses anteriormente utilizadas foram otimizadas até a dose máxima tolerada. Ao final desse período, os pacientes que permaneceram sintomáticos foram randomizados (1:1) eletronicamente usando software para randomização permutada em blocos para receber 1 dos seguintes medicamentos: trimetazidina (35 mg duas vezes ao dia) ou alopurinol (300 mg duas vezes ao dia). Durante o período do estudo, outros medicamentos não foram introduzidos e as doses não foram ajustadas.

Os pacientes não foram cegados quanto ao grupo de intervenção no qual foram alocados, mas o pesquisador responsável pela avaliação da angina e aplicação dos questionários estava cego quanto à intervenção.

### Avaliação da angina

Os pacientes foram avaliados 30 dias após o início da terapia designada. O desfecho primário avaliado foi a diferença na pontuação média do Questionário de Angina de Seattle (QAS) no domínio da frequência da angina (QAS-FA) 30 dias após o início do tratamento.

O questionário possui 19 itens que avaliam 5 domínios do estado de saúde relacionados à DAC, com pontuações que variam de 0 a 100; pontuações mais altas indicam menos sintomas e melhor estado de saúde.^9 , 10^

Os desfechos secundários avaliados foram a diferença na pontuação total obtida para os 5 domínios do QAS (QAS total) em 30 dias; número de episódios semanais de angina; quantidade de nitroglicerina sublingual de curta ação utilizada semanalmente; e qualidade de vida de acordo com a pontuação do Medical Outcomes Study 36-Item Short Form Health Survey (SF-36), no seguimento de 30 dias.

O questionário SF-36 é composto por 36 itens correspondentes a 8 domínios; pontuações mais altas indicam melhor percepção de saúde, função preservada e ausência de dor.

### Tamanho amostral

O tamanho amostral foi calculado a partir de um estudo anterior que incluiu pacientes com angina estável e utilizou a pontuação do QAS-FA como desfecho.^10^ Uma amostra de 108 pacientes (54 pacientes em cada braço de tratamento do estudo) foi calculada para observar uma diferença de 20% entre grupos no desfecho primário, estimando poder de estudo de 80% e erro alfa de 5%.

### Aspectos éticos

O presente estudo foi aprovado pelo comitê de ética da instituição onde foi realizado (CAAE: 93752618.9.0000.0045) e está registrado no Registro Brasileiro de Ensaios Clínicos (Número de Registro RBR-5kh98y). Todos os procedimentos foram realizados de acordo com a Declaração de Helsinque.

### Análise estatística

O teste de Kolmogorov–Smirnov foi utilizado para verificar a distribuição normal das variáveis contínuas. As variáveis com distribuição normal são apresentadas como média e desvio padrão (DP), e os dados com distribuição assimétrica são apresentados como mediana e percentis 25 e 75. As variáveis categóricas são apresentadas como frequência e porcentagem. As comparações das variáveis categóricas foram realizadas por meio do teste do qui-quadrado. A comparação das pontuações dos domínios entre a linha de base e o seguimento foi realizada por meio do teste t pareado para variáveis com distribuição paramétrica e do teste de Wilcoxon para aquelas com distribuição não paramétrica. A comparação da diferença nos escores dos domínios entre os grupos de intervenção no seguimento foi realizada por meio do teste t de amostras independentes para variáveis com distribuição paramétrica e do teste de Mann–Whitney para aquelas com distribuição não paramétrica. Foram considerados estatisticamente significativos valores de probabilidade (p) < 0,05. Foi utilizado o Statistical Package for the Social Sciences (SPSS) versão 20.0 para análise dos dados.

## Resultados

**Figura 1:**
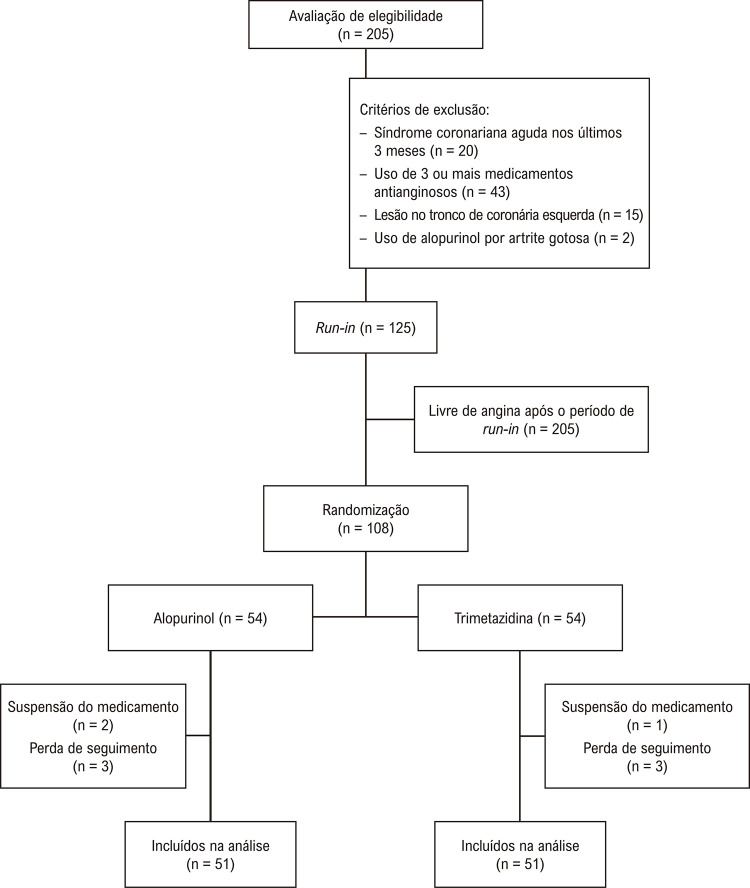
– Fluxograma dos pacientes avaliados e incluídos no run-in, randomização e seguimento.

Um total de 205 pacientes com DAC e angina foram avaliados para inclusão no estudo, 125 (61%) dos quais foram incluídos na fase inicial; os demais foram excluídos por já estarem em uso de 3 ou mais antianginosos, apresentarem SCA há menos de 3 meses, obstrução de tronco de coronária esquerda ≥ 50% ou indicação de uso de alopurinol por artrite gotosa ( Figura 1 ). Após um período mínimo de 1 semana em uso de betabloqueadores e bloqueadores dos canais de cálcio em doses otimizadas, 17 (13,6%) indivíduos estavam livres de sintomas de angina. Os 108 pacientes restantes foram incluídos na fase de randomização, sendo 54 (50%) randomizados para o grupo alopurinol e 54 (50%) para o grupo trimetazidina. Seis (5,6%) indivíduos, 3 de cada grupo, foram perdidos no seguimento para o desfecho primário. Três (2,8%) pacientes descontinuaram o uso da medicação durante o seguimento, sendo 2 do grupo alopurinol e 1 do grupo trimetazidina. O motivo da descontinuação foram efeitos colaterais menores envolvendo o trato gastrointestinal.

As características de linha de base dos pacientes são apresentadas na Tabela 1 . A média de idade foi de 60,2 ± 8,6 anos; 60 (55,6%) participantes eram do sexo masculino; 100 (93,5%) participantes foram diagnosticados com hipertensão, 62 (57,9%) com diabetes mellitus, 54 (50,0%) com SCA no último ano e 40 (37,0%) participantes foram submetidos a revascularização miocárdica cirúrgica ou percutânea. Não houve diferença nas características basais entre os grupos.

**Tabela 1:** – Características basais e apresentação clínica

	Alopurinol N = 54	Trimetazidina N = 54	p
**Sexo masculino, n (%)**	32 (59,3%)	28 (51,9%)	0,562
**Idade (anos), média ± DP**	60,3 ±8,1	60,1 ±9,2	0,912
**Comorbidades**			
Hipertensão arterial sistêmica, n (%)	50 (92,6%)	50 (92,6%)	0,999
Diabetes mellitus, n (%)	31 (57,4%)	31 (57,4%)	0,999
AVC, n (%)	5 (9,4%)	2 (3,7%)	0,270
SCA no último ano, n (%)	15 (27,8%)	16 (29,6%)	0,999
Revascularização miocárdica prévia, n (%)	17 (31,5%)	23 (42,6%)	0,411
FEVE, média ± DP	58,8 ±11,7	61,5 ±11,2	0,243
**Apresentação clínica**			
Angina, CCS III/IV, n (%)	26 (48,1%)	21 (38,9%)	0,554
FC (bpm), média ± DP	72,2 ±10,6	72,4 ±12,2	0,908
PAS (mmHg), média ± DP	133,3 ±19,3	133,8 ±22,1	0,906
Isquemia na SPECT (%), média ± DP	8,5 ±10,9	6,5 ±7,4	0,413
Artéria coronária com obstrução ≥ 70%, média ± DP	2,1 ±0,7	2,2 ±0,8	0,575
**Medicamentos**			
AAS, n (%)	54 (100%)	54 (100%)	- -
Estatina, n (%)	53 (98,1%)	54 (100%)	0,999
IECA/BRA, n (%)	50 (96,6%)	51 (94,4%)	0,999

AAS: ácido acetilsalicílico; AVC: acidente vascular cerebral; BRA: bloqueador dos receptores da angiotensina; CCS: classificação da Canadian Cardiovascular Society; DP: desvio padrão; DRC: doença renal crônica; FC: frequência cardíaca; FEVE: fração de ejeção do ventrículo esquerdo; IECA: inibidor da enzima conversora de angiotensina; PAS: pressão arterial sistólica; SCA: síndrome coronariana aguda; SPECT: tomografia computadorizada por emissão de fóton único.

Angina grau III/IV da Canadian Cardiovascular Society estava presente em 47 (43,5%) participantes na avaliação inicial. A mediana da pontuação do QAS-FA foi 50 (20 a 70) e a média da pontuação do QAS total foi 42,4 ± 19,1. Nos grupos alopurinol e trimetazidina, as medianas da pontuação do QAS-FA foram 50 (30 a 70) e 50 (21,3 a 78,3), respectivamente, e as médias da pontuação do QAS total foram 43,5 ± 18,5 e 41,4 ± 20,0, respectivamente.

Em ambos os grupos, a pontuação para todos os domínios melhorou em relação à linha de base, exceto a satisfação com o tratamento no grupo alopurinol ( Tabela 2 ).

**Tabela 2:** – Efeito do alopurinol e da trimetazidina nas pontuações dos domínios do Questionário de Angina de Seattle (QAS), episódios semanais de angina

	Alopurinol			Trimetazidina		
	
	Linha de base	Seguimento	p	Linha de base	Seguimento	p
Limitação física, mediana (25 a 75)	38,9 (27,1-53,5)	44,4 (30,6-78,4)	<0,001*	38,9 (27,8-52,8)	55,5 (39,2-77,1)	<0,001 *
Estabilidade da angina, mediana (25 a 75)	50,0 (25,0-75,0)	75,0 (50,0-100,0)	0,027 *	50,0 (6,25-75,0)	87,5 (75,0-100,0)	<0,001 *
Frequência da angina, mediana (25 a 75)	50,0 (30,0-70,0)	65,0 (47,5-80)	<0,001 *	50,0 (21,3-73,8)	80,0 60,0-90,0	<0,001 *
Satisfação com o tratamento, mediana (25 a 75)	87,5 (73,5-100,0)	93,8 (73,5-100,0)	0,602 *	87,5 (75,0-100,0)	93,8 (81,3-100,0)	0,018 *
Percepção da doença, mediana (25 a 75)	41,7 (25,0-60,4)	58,3 (33,3-75,0)	0,001 *	33,3 (25,0-64,6)	62,2 (33,3-89,6)	<0,001 *
QAS total, média ± DP	43,7 ± 18,5	56,5 ± 22,3	<0,001 †	42,7 ± 19,7	63,9 ± 23,1	<0,001 †
Episódios de angina/semana, mediana (25 a 75)	5 (3-7)	4 (3-7)	<0,001	3 (1-5,5)	2 (0,9-3)	<0,001*

* Teste de Wilcoxon; † teste t de amostras dependentes. QAS: Questionário de Angina de Seattle.

**Figura 2:**
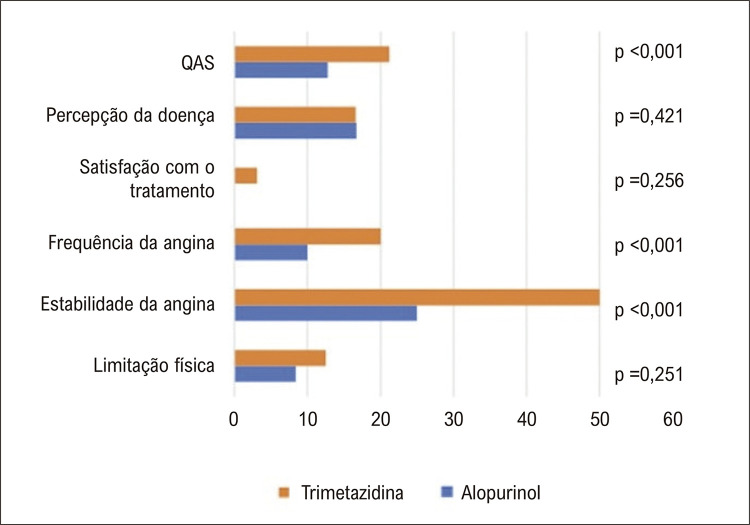
– Diferença nos domínios do Questionário de Angina de Seattle (QAS) em relação à linha de base nos grupos alopurinol e trimetazidina.

A diferença mediana em relação à linha de base para a pontuação do QAS-FA foi menor no grupo alopurinol (10 [0 a 30] versus 20 [10 a 40]; p < 0,001), assim como a diferença média no escore QAS total (12,8 ± 17,8 versus 21,2 ± 15,9; p = 0,014). Também foi observada diferença no domínio de estabilidade ( Figura 2 ). Tanto o alopurinol quanto a trimetazidina reduziram os episódios semanais de angina ( Tabela 2 ).

Na avaliação da qualidade de vida do SF-36, o grupo alopurinol melhorou apenas no domínio de aspecto físico, e o grupo trimetazidina melhorou em todos os domínios, exceto vitalidade e estado geral de saúde ( Tabela 3 ).

**Tabela 3:** – Efeito do alopurinol e da trimetazidina nas pontuações dos domínios do SF-36

	Alopurinol			Trimetazidina		
	
	Linha de base	Seguimento	p *	Linha de base	Seguimento	p *
Capacidade funcional, mediana	35 (15-55)	35 (25-59)	0,374	35 (20-50)	50 (35-75)	0,003
Aparência física, mediana	25 (0-25)	25 (25-75)	0,015	25 (0-25)	25 (0-75)	0,002
Aspectos emocionais, mediana	67 (33-100)	67 (33-100)	0,766	33 (0-100)	67 (33-100)	0,022
Vitalidade, mediana	55 (35-70)	50 (30-69)	0,880	45 (15-75)	55 (30-70)	0,163
Saúde mental, mediana	66 (37-84)	64 (37-84)	0,722	52 (48-76)	72 (48-84)	0,049
Aspectos sociais, mediana	75 (41-100)	75 (25-100)	0,837	63 (50-100)	88 (50-100)	0,019
Dor, mediana	35 (23-47)	55 (33-70)	0,074	33 (23-45)	55 (33-70)	<0,001
Estado geral de saúde, mediana	47 (31-62)	52 (41-72)	0,163	45 (32-67)	52 (30-77)	0,305

* Teste de Wilcoxon.

Não houve efeitos colaterais graves em nenhum dos pacientes incluídos. Sete (6,5%) indivíduos apresentaram náuseas, vômitos ou distensão abdominal, 4 com alopurinol e 3 com trimetazidina. Desses participantes, 3 descontinuaram o uso da medicação por causa dos sintomas: 2 no grupo alopurinol e 1 no grupo trimetazidina. Todos os pacientes apresentaram resolução dos sintomas ao longo do seguimento.

## Discussão

No estudo ATTRACT, o alopurinol e a trimetazidina melhoraram os sintomas de angina, avaliados pela pontuação QAS; no entanto, a trimetazidina apresentou um ganho maior em relação à linha de base. A diferença ocorreu devido a melhorias mais significativas nos domínios de frequência e estabilidade.

Este é um dos poucos ensaios clínicos que comparou 2 antianginosos com mecanismos metabólicos e que avaliou o efeito do alopurinol na angina.^11 , 12^ Ambos são medicamentos amplamente utilizados com perfis de segurança satisfatórios; notavelmente, o alopurinol é uma terapia de baixo custo que tem mostrado resultados promissores em um estudo anterior.

Vários ensaios clínicos demonstraram que não há superioridade entre tratamentos intervencionistas e tratamento cirúrgico ou percutâneo em pacientes com DAC estável em relação a desfechos cardiovasculares maiores (morte e infarto agudo do miocárdio).^1 , 2 , 13^ No entanto, faltam ensaios clínicos que avaliem a eficácia de agentes antianginosos.^11^

Em um cenário de aumento da expectativa de vida de indivíduos com DAC e de ainda maior relevância da terapia clínica, é de extrema importância a realização de estudos como este, que visem melhorar o tratamento clínico para o controle dos sintomas anginosos e aumentar a qualidade de vida.

Durante a fase de *run-in* , a maioria dos indivíduos já estava em uso de betabloqueadores e/ou bloqueadores dos canais de cálcio, embora em doses não otimizadas. Mesmo após a otimização do tratamento, apenas 15% dos indivíduos permaneceram livres de angina. Esses dados reforçam a dificuldade em controlar esse sintoma na DAC e a necessidade de estudos que avaliem combinações de diferentes classes de medicamentos antianginosos.

Em nosso estudo, o tratamento com alopurinol resultou em melhora de 10 pontos na pontuação do QAS-FA, e o tratamento com trimetazidina levou a uma melhora de 20 pontos. Estudos anteriores relataram melhorias de 17 pontos com ranolazina,^14^ 12 pontos com atenolol,^15^ 14 pontos com carvedilol,^15^ 12 pontos com angioplastia em pacientes com obstrução crônica^16^ e 11 pontos com angioplastia no estudo ORBITA.^17^

A trimetazidina deve continuar a ser um agente antianginoso metabólico de primeira linha, dada sua superioridade na redução dos sintomas anginosos. Porém, com resultados satisfatórios, o alopurinol é uma opção econômica para o controle da angina, principalmente em países em desenvolvimento, pois seu custo proporcional a cada ponto de redução na pontuação do QAS-FA é inferior ao da trimetazidina. No contexto atual, a relação custo-eficácia das intervenções de saúde deve ser cada vez mais valorizada.

Os indivíduos do grupo alopurinol não apresentaram diferença na qualidade de vida após o tratamento de acordo com o escore SF-36, apesar de apresentarem melhora no domínio de qualidade de vida do QAS. O SF-36, por ser um instrumento amplo, não é específico para avaliação de pacientes com DAC; o QAS é mais específico para esta doença.^18 , 19^ Assim, é possível que as melhorias na qualidade de vida após o uso de alopurinol tenham sido mais aparentes com base em uma escala relacionada à doença específica e que o uso de trimetazidina tenha levado a melhorias de um aspecto mais amplo da saúde em geral.

### Limitações do estudo

Os fatores limitantes do presente estudo foram a ausência de um grupo placebo e o não cegamento dos indivíduos randomizados quanto à intervenção. Na ausência de um grupo placebo, as melhorias atribuídas ao uso de alopurinol podem ser explicadas como um possível efeito placebo. Entretanto, em estudos anteriores que avaliaram os escores do QAS-FA em grupos placebo randomizados, houve um aumento de aproximadamente 1,6 a 7,7 pontos.^17 , 20^ Assim, a magnitude do efeito encontrada para o grupo alopurinol não é consistente com o efeito placebo. Embora os pacientes não estivessem cegos, os pesquisadores que avaliaram os pacientes e aplicaram o questionário ficaram cegos quanto à intervenção, reduzindo a possibilidade de viés.

## Conclusões

Tanto o alopurinol quanto a trimetazidina melhoraram o controle dos sintomas de angina; no entanto, a trimetazidina levou a ganhos maiores em relação à linha de base. Portanto, ambos são opções terapêuticas como antianginosos, e a trimetazidina deve continuar sendo a opção de primeira linha entre os medicamentos metabólicos.
