# Supplementary material for: Alopurinol Versus Trimetazidina para o Tratamento da Angina: Ensaio Clínico Randomizado
Source: Arq Bras Cardiol. 2024 Jul 25;121(8):e20230659. [Article in Portuguese] doi: 10.36660/abc.20230659 (PMC11463339; doi:10.36660/abc.20230659)
Supplement: Translation

# Translation: Allopurinol versus Trimetazidine for the Treatment of Angina: A Randomized Clinical Trial

**DOI:** 10.36660/abc.20230659i

**Authors:** Tainá Viana, Rodrigo Morel Vieira de Melo, Diogo Freitas Cardoso Azevedo, Clara Salles Figueiredo, Gustavo Santana, Luanna Mota Damasceno, Luisa Latado, Ludmila Tambuque, Raissa Barreto, Luiz Carlos Santana Passos

**Affiliations:** 1 Brazil Universidade Federal da Bahia, Salvador, BA – Brazil; 2 Brazil Ana Nery Hospital, Salvador, BA – Brazil

**Keywords:** Alopurinol, Trimetazidine, Doença Myocardial Ischemia, Angina Pectoris

## Abstract

**Background:**

Recently, it was demonstrated that allopurinol, a xanthine oxidase inhibitor, has cardiovascular and anti-ischaemic properties and may be a metabolic antianginal agent option.Objective: The objective of this study was to evaluate the antianginal effect of allopurinol as a third drug for patients with stable coronary artery disease (CAD).

**Methods:**

This was a randomized clinical trial between 2018 and 2020 including patients with CAD who maintained angina despite initial optimization with beta-blockers and calcium channel blockers. The individuals were randomized 1:1 to 300 mg of allopurinol twice daily or 35 mg of trimetazidine twice daily. The main outcome was the difference in the angina frequency domain of the Seattle Angina Questionnaire (SAQ-AF). A probability (p) value < 0.05 was considered statistically significant.

**Results:**

A hundred and eight patients were included in the randomization phase, with 54 (50%) in the allopurinol group and 54 (50%) in the trimetazidine group. Six (5.6%) individuals, 3 from each group, were lost to follow-up for the primary outcome. In the allopurinol and trimetazidine groups, the median SAQ-AF scores were 50 (30.0 to 70.0) and 50 (21.3 to 78.3), respectively. In both groups, the SAQ-AF score improved, but the median of the difference compared to baseline was lower in the allopurinol group (10 [0 to 30] versus 20 [10 to 40]; p < 0.001), as was the mean of the difference in the total SAQ score (12.8 ± 17.8 versus 21.2 ± 15.9; p = 0.014).

**Conclusion:**

Both allopurinol and trimetazidine improved the control of angina symptoms; however, trimetazidine presented a greater gain compared to baseline. **Brazilian Registry of Clinical Trials - Registration Number RBR-5kh98y**

**Central Illustration:** : Allopurinol versus Trimetazidine for the Treatment of Angina: A Randomized Clinical Trial

Allopurinol and trimetazidine improved the control of angina symptoms; however, trimetazadine led to greater gains compared to baseline. BB: beta-blocker; BID: twice daily; CCB: calcium channel blocker; SAQ: Seattle Angina Questionnaire.

## Introduction

With advancements in the treatment of risk factors for atherosclerotic disease, several studies have demonstrated the effectiveness of clinical treatments as the initial choice for coronary artery disease (CAD).^1 , 2^The priorities in this context are the control of risk factors and symptomatic improvements in angina.^3^

Current guidelines recommend the use of beta-blockers and calcium channel blockers as the initial drugs for relieving angina symptoms.^3^Second-line agents include trimetazidine, ivabradine, and long-acting nitrates. Allopurinol, a xanthine oxidase inhibitor, has demonstrated cardiovascular and anti-ischaemic properties.^4 - 7^In a previous study, 300 mg of allopurinol twice daily increased the time to ST-segment depression and the total time in an exercise stress test.^8^

However, studies that evaluated the effect of allopurinol on CAD used laboratory outcomes or changes in diagnostic tests as the primary outcome.^4 - 8^The prevalence of angina or cardiovascular events was evaluated only as secondary outcomes; therefore, it is necessary to conduct studies that evaluate, in a primary and systematic manner, the medication’s effect on angina, the symptom with the greatest impact on patients with stable CAD.

The objective of the ATTRACT study (Allopurinol versus Trimetazidine as a Third Drug for the Treatment of Angina: a Randomized Clinical Trial) is to compare allopurinol versus trimetazidine as a third drug to control angina in patients with CAD and stable angina refractory to the maximum tolerated doses of beta-blockers and calcium channel blockers.

## Methods

This was a randomized, single-centre clinical trial that included patients with symptoms of angina from a specialized CAD outpatient clinic between 2018 and 2020.

### Patients

Patients older than 18 years of age with stable CAD diagnosed through cardiac catheterization that revealed at least 1 epicardial coronary artery with stenosis greater than 70% treated at the outpatient clinical of a referral hospital were asked to participate. Coronary angiography was performed in the context of previous acute coronary syndrome (ACS) or in the context of stable coronary disease with a high probability of CAD or persistent symptoms. All patients gave written informed consent.

Patients received clinical treatment optimized with a beta-blocking agent at the maximum tolerated dose and a dihydropyridine calcium channel antagonist.

The exclusion criteria were ACS in the last 3 months, scheduled surgical or percutaneous myocardial revascularization, obstruction in the left main coronary artery > 50%, asymptomatic angina after initial clinical optimization, hepatocellular dysfunction, chronic kidney disease with creatinine clearance less than 30 ml/min/1.73 m^2^, gouty arthritis that warranted the use of allopurinol, and refusal to participate in the study and/or sign the informed consent form.

### Trial procedures

The patients underwent a minimum of 1-week run-in period with a beta-blocking agent combined with a dihydropyridine-type calcium channel antagonist at optimized doses. The drugs were added or, when the medication was already used, the previously used doses were optimized until the maximum tolerated dose. At the end of this period, patients who remained symptomatic were randomized (1:1) electronically using software for block-permuted randomization to receive 1 of the following medications: trimetazidine (35 mg twice daily) or allopurinol (300 mg twice daily). During the study period, other medications were not introduced, and doses were not adjusted.

The patients were not blinded to the intervention group they were allocated to, but the researcher responsible for assessing angina and applying the questionnaires was blinded to the intervention.

### Evaluation of angina

The patients were evaluated 30 days after the beginning of the designated therapy. The primary outcome evaluated was the difference in the mean score on the Seattle Angina Questionnaire (SAQ) in the angina frequency domain (SAQ-AF) 30 days after the beginning of treatment.

The questionnaire has 19 items that measure 5 health status domains related to CAD, with scores ranging from 0 to 100; higher scores indicate fewer symptoms and a better health status.^9 , 10^

The secondary outcomes evaluated were the difference in the total score obtained for the 5 SAQ domains (total SAQ) at 30 days; number of weekly episodes of angina; amount of short-acting sublingual nitroglycerin used weekly; and quality of life according to Medical Outcomes Study 36-Item Short Form Health Survey (SF-36) score, at the 30-day follow-up.

The SF-36 questionnaire consists of 36 items corresponding to 8 domains; higher scores indicate better perception of health, preserved function, and absence of pain.

### Sample size

The sample size was calculated from a previous study that included patients with stable angina and used the SAQ-AF score as an outcome.^10^A sample of 108 patients (54 patients in each treatment arm of the study) was calculated to observe a difference of 20% between groups in the primary outcome, estimating a study power of 80% and alpha error of 5%.

### Ethical aspects

This study was approved by the ethics committee of the institution where it was conducted (CAAE: 93752618.9.0000.0045), and it is registered in the Brazilian Registry of Clinical Trials (Registration Number RBR-5kh98y). All procedures were performed in accordance with the Declaration of Helsinki.

### Statistical analysis

The Kolmogorov–Smirnov test was used to verify the normal distribution of continuous variables. Variables with a normal distribution are reported as means and standard deviations (SD), and data with a nonsymmetric distribution are reported as medians and 25th and 75th percentiles. Categorical variables are reported as frequencies and percentages. Comparisons of categorical variables were performed using the chi-square test. The comparison of domain scores between baseline and follow-up was performed using the paired t test for variables with a parametric distribution and the Wilcoxon test for those with a nonparametric distribution. The comparison of the difference in domain scores between the intervention groups at follow-up was performed using the t test of independent samples for variables with a parametric distribution and the Mann–Whitney test for those with a nonparametric distribution. A probability (p) value < 0.05 was considered statistically significant. The Statistical Package for the Social Sciences (SPSS) version 20.0 was used for data analysis.

## Results

A total of 205 patients with CAD and angina were evaluated for inclusion in the study, 125 (61%) of whom were included in the run-in phase; the others were excluded because they were already using 3 or more antianginal drugs, presented ACS in less than 3 months, left main coronary obstruction ≥ 50%, or indications for the use of allopurinol due to gouty arthritis ( Figure 1 ). After a minimum period of 1 week using beta-blockers and calcium channel blockers at optimized doses, 17 (13.6%) individuals were free of angina symptoms. The remaining 108 patients were included in the randomization phase, with 54 (50%) randomized to the allopurinol group and 54 (50%) to the trimetazidine group. Six (5.6%) individuals, 3 from each group, were lost to follow-up for the primary outcome. Three (2.8%) patients discontinued the use of the medication during follow-up, 2 from the allopurinol group and 1 from the trimetazidine group. The reason for discontinuation was minor side effects involving the gastrointestinal tract.

**Figure 1:** – Flowchart of the patients evaluated and included in the run-in, randomization, and follow-up.

The baseline characteristics of the patients are provided in Table 1 . The mean age was 60.2 ± 8.6 years; 60 (55.6%) participants were male; 100 (93.5%) participants were diagnosed with hypertension, 62 (57.9%) with diabetes mellitus, 54 (50.0%) with ACS in the past year, and 40 (37.0%) participants underwent surgical or percutaneous myocardial revascularization. There was no difference in baseline characteristics between the groups.

**Table 1:** – Baseline characteristics and clinical presentation

	Allopurinol N = 54	Trimetazidine N = 54	p
**Male, n (%)**	32 (59.3%)	28 (51.9%)	0.562
**Age (years), mean ± SD**	60.3 ±8.1	60.1 ±9.2	0.912
**Comorbidities**			
Systemic arterial hypertension, n (%)	50 (92.6%)	50 (92.6%)	0.999
Diabetes mellitus, n (%)	31 (57.4%)	31 (57.4%)	0.999
Stroke, n (%)	5 (9.4%)	2 (3.7%)	0.270
ACS in the last year, n (%)	15 (27.8%)	16 (29.6%)	0.999
Previous myocardial revascularization, n (%)	17 (31.5%)	23 (42.6%)	0.411
LVEF, mean ± SD	58.8 ±11.7	61.5 ±11.2	0.243
**Clinical presentation**			
Angina, CCS III/IV, n (%)	26 (48.1%)	21 (38.9%)	0.554
HR (bpm), mean ± SD	72.2 ±10.6	72.4 ±12.2	0.908
SBP (mmHg), mean ± SD	133.3 ±19.3	133.8 ±22.1	0.906
Ischemia in SPECT (%), mean ± SD	8.5 ±10.9	6.5 ±7.4	0.413
Coronary artery with obstruction ≥ 70%, mean ±SD	2.1 ±0.7	2.2 ±0.8	0.575
**Medications**			
ASA, n (%)	54 (100%)	54 (100%)	- -
Statin, n (%)	53 (98.1%)	54 (100%)	0.999
ACEI/ARB, n (%)	50 (96.6%)	51 (94.4%)	0.999

ACEI: angiotensin-converting enzyme inhibitor; ACS: acute coronary syndrome; ARB: angiotensin receptor blocker; ASA: acetylsalicylic acid; CCS: Canadian Cardiovascular Society grading; CKD: chronic kidney disease; HR: heart rate; LVEF: left ventricular ejection fraction; SBP: systolic blood pressure; SD: standard deviation; SPECT: single-photon emission computed tomography.

Canadian Cardiovascular Society angina grade III/IV of was present in 47 (43.5%) participants at the initial evaluation; the median SAQ-AF score was 50 (20 to 70), and the mean total SAQ score was 42.4 ± 19.1. In the allopurinol and trimetazidine groups, the median SAQ-AF scores were 50 (30 to 70) and 50 (21.3 to 78.3), respectively, and the mean total SAQ scores were 43.5 ± 18.5 and 41.4 ± 20.0, respectively.

In both groups, score for all domains improved compared with baseline, except satisfaction with treatment in the allopurinol group ( Table 2 ).

**Table 2:** – Effect of allopurinol and trimetazidine on Seattle Angina Questionnaire (SAQ) domain scores, weekly episodes of angina

	Allopurinol			Trimetazidine		
	
	Baseline	Follow-up	p	Baseline	Follow-up	p
Physical limitation, median (25th - 75th)	38.9 (27.1-53.5)	44.4 (30.6-78.4)	<0.001*	38.9 (27.8-52.8)	55.5 (39.2-77.1)	<0.001 *
Stability of angina, median (25th - 75th)	50.0 (25.0-75.0)	75.0 (50.0-100.0)	0.027 *	50.0 (6.25-75.0)	87.5 (75.0-100.0)	<0.001 *
Frequency of angina, median (25th - 75th)	50.0 (30.0-70.0)	65.0 (47.5-80)	<0.001 *	50.0 (21.3-73.8)	80.0 60.0-90.0	<0.001 *
Satisfaction with treatment, median (25th - 75th)	87.5 (73.5-100.0)	93.8 (73.5-100.0)	0.602 *	87.5 (75.0-100.0)	93.8 (81.3-100.0)	0.018 *
Perception of disease, median (25th - 75th)	41.7 (25.0-60.4)	58.3 (33.3-75.0)	0.001 *	33.3 (25.0-64.6)	62.2 (33.3-89.6)	<0.001 *
SAQ-total, mean ± SD	43.7 ± 18.5	56.5 ± 22.3	<0.001 †	42.7 ± 19.7	63.9 ± 23.1	<0.001 †
Angina episodes/week, median (25th - 75th)	5 (3-7)	4 (3-7)	<0.001	3 (1-5.5)	2 (0.9-3)	<0.001*

* Wilcoxon test; † t test of dependent samples. SAQ: Seattle Angina Questionnaire.

The median difference from baseline for the SAQ-AF score was lower in the allopurinol group (10 [0 to 30] versus 20 [10 to 40]; p < 0.001), as was the mean difference in the total SAQ score (12.8 ± 17.8 versus 21.2 ± 15.9; p = 0.014). A difference was also observed in the stability domain ( Figure 2 ). Both allopurinol and trimetazidine reduced the weekly episodes of angina ( Table 2 ).

**Figure 2:** – Difference in Seattle Angina Questionnaire (SAQ) domains compared to baseline in the allopurinol and trimetazidine groups.

In the SF-36 quality of life assessment, the allopurinol group improved only in the physical aspect domain, and the trimetazidine group improved in all domains, except vitality and general health status ( Table 3 ).

**Table 3:** – Effect of allopurinol and trimetazidine on SF-36 domain scores

	Allopurinol			Trimetazidine		
	
	Baseline	Follow-up	p *	Baseline	Follow-up	p *
Functional capacity, median	35 (15-55)	35 (25-59)	0.374	35 (20-50)	50 (35-75)	0.003
Physical appearance, median	25 (0-25)	25 (25-75)	0.015	25 (0-25)	25 (0-75)	0.002
Emotional aspects, median	67 (33-100)	67 (33-100)	0.766	33 (0-100)	67 (33-100)	0.022
Vitality, median	55 (35-70)	50 (30-69)	0.880	45 (15-75)	55 (30-70)	0.163
Mental health, median	66 (37-84)	64 (37-84)	0.722	52 (48-76)	72 (48-84)	0.049
Social aspects, median	75 (41-100)	75 (25-100)	0.837	63 (50-100)	88 (50-100)	0.019
Pain, median	35 (23-47)	55 (33-70)	0.074	33 (23-45)	55 (33-70)	<0.001
General health status, median	47 (31-62)	52 (41-72)	0.163	45 (32-67)	52 (30-77)	0.305

* Wilcoxon test.

There were no serious side effects in any of the included patients. Seven (6.5%) individuals had nausea, vomiting or bloating, 4 with allopurinol and 3 with trimetazidine; of these participants, 3 discontinued the use of the medication because of the symptoms: 2 in the allopurinol group and 1 in the trimetazidine group. All patients exhibited resolution of symptoms throughout follow-up.

## Discussion

In the ATTRACT study, allopurinol and trimetazidine imroved angina symptoms, as assessed by the SAQ score; however, trimetazidine presented greater gains compared to baseline. The difference was due to more significant improvements in the frequency and stability domains.

This is one of the few clinical trials that has compared 2 antianginals with metabolic mechanisms and that has evaluated the effect of allopurinol on angina.^11 , 12^Both are widely used medications with satisfactory safety profiles; notably, allopurinol is a low-cost therapy that has shown promising results in a previous study.

Several clinical trials have shown that there is no superiority between interventional treatments and surgical or percutaneous treatment in patients with stable CAD for major cardiovascular outcomes (death and acute myocardial infarction).^1 , 2 , 13^However, there is a lack of clinical trials that have evaluated the efficacy of antianginal agents.^11^

In a scenario of increased life expectancy of individuals with CAD and even greater relevance of clinical therapy, it is extremely important to conduct studies, such as this one, that aim to improve clinical treatment for the control of anginal symptoms and increase quality of life.

In the run-in phase, most individuals were already using beta-blockers and/or calcium channel blockers, albeit in non-optimized doses. Even after optimizing treatment, only 15% of individuals remained angina-free. These data reinforce the difficulty of controlling this symptom in CAD and the need for studies evaluating combinations of different classes of anti-anginal drugs.

In our study, treatment with allopurinol resulted in a 10-point improvement in the SAQ-AF score, and treatment with trimetazidine led to a 20-point improvement. Previous studies reported improvements of 17 points with ranolazine,^14^12 points with atenolol,^15^14 points with carvedilol,^15^12 points with angioplasty in chronic obstruction,^16^and 11 points with angioplasty in the ORBITA study.^17^

Trimetazidine should remain a first-line metabolic antianginal agent, given its superiority in reducing anginal symptoms. However, with satisfactory results, allopurinol is an inexpensive option for the control of angina, especially in developing countries, because its cost proportional to each point of reduction in the SAQ-AF score is lower than that of trimetazidine. In the current context, the cost-effectiveness of health interventions should be increasingly valued.

Individuals in the allopurinol group showed no difference in quality of life after treatment according to the SF-36 score, despite showing improvements in the SAQ quality of life domain. The SF-36, as a broad instrument, is not specific for the evaluation of patients with CAD; the SAQ is more specific for this disease.^18 , 19^Thus, it is possible that the improvements in quality of life after the use of allopurinol were more apparent based on a scale related to the specific disease and that the use of trimetazidine led to improvements from a broader aspect of health in general.

### Study limitations

The limiting factors of the present study were the lack of a placebo group and nonblinding to the intervention for randomized individuals. In the absence of a placebo group, the improvements attributed to the use of allopurinol can be explained as a possible placebo effect. However, in previous studies that evaluated SAQ-AF scores in randomized placebo groups, there was an increase of approximately 1.6 to 7.7 points.^17 , 20^Thus, the magnitude of the effect found for the allopurinol group is not consistent with the placebo effect. Even though the patients were not blinded, the researchers who evaluated the patients and applied the questionnaire were blinded to the intervention, reducing the possibility of bias.

## Conclusions

Both allopurinol and trimetazidine improved the control of angina symptoms; however, trimetazidine led to greater gains compared to baseline. Therefore, both are therapeutic options as antianginal drugs, and trimetazidine should remain the first-line option among metabolic drugs.
